# Cryo-EM structures for the *Mycobacterium tuberculosis* iron-loaded siderophore transporter IrtAB

**DOI:** 10.1093/procel/pwac060

**Published:** 2022-11-17

**Authors:** Shan Sun, Yan Gao, Xiaolin Yang, Xiuna Yang, Tianyu Hu, Jingxi Liang, Zhiqi Xiong, Yuting Ran, Pengxuan Ren, Fang Bai, Luke W Guddat, Haitao Yang, Zihe Rao, Bing Zhang

**Affiliations:** Shanghai Institute for Advanced Immunochemical Studies and School of Life Science and Technology, ShanghaiTech University, Shanghai 201210, China; CAS Center for Excellence in Molecular Cell Science, Shanghai Institute of Biochemistry and Cell Biology, Chinese Academy of Sciences, Shanghai 200031, China; University of Chinese Academy of Sciences, Beijing 100101, China; Shanghai Institute for Advanced Immunochemical Studies and School of Life Science and Technology, ShanghaiTech University, Shanghai 201210, China; Shanghai Institute for Advanced Immunochemical Studies and School of Life Science and Technology, ShanghaiTech University, Shanghai 201210, China; Shanghai Institute for Advanced Immunochemical Studies and School of Life Science and Technology, ShanghaiTech University, Shanghai 201210, China; Shanghai Clinical Research and Trial Center, Shanghai 201210, China; Shanghai Institute for Advanced Immunochemical Studies and School of Life Science and Technology, ShanghaiTech University, Shanghai 201210, China; State Key Laboratory of Medicinal Chemical Biology, Nankai University, Tianjin 300353, China; Laboratory of Structural Biology, Tsinghua University, Beijing 100084, China; Shanghai Institute for Advanced Immunochemical Studies and School of Life Science and Technology, ShanghaiTech University, Shanghai 201210, China; Shanghai Institute for Advanced Immunochemical Studies and School of Life Science and Technology, ShanghaiTech University, Shanghai 201210, China; Shanghai Institute for Advanced Immunochemical Studies and School of Life Science and Technology, ShanghaiTech University, Shanghai 201210, China; School of Chemistry and Molecular Biosciences, The University of Queensland, Brisbane, Queensland 4072, Australia; Shanghai Institute for Advanced Immunochemical Studies and School of Life Science and Technology, ShanghaiTech University, Shanghai 201210, China; Shanghai Clinical Research and Trial Center, Shanghai 201210, China; Shanghai Institute for Advanced Immunochemical Studies and School of Life Science and Technology, ShanghaiTech University, Shanghai 201210, China; State Key Laboratory of Medicinal Chemical Biology, Nankai University, Tianjin 300353, China; Laboratory of Structural Biology, Tsinghua University, Beijing 100084, China; National Laboratory of Biomacromolecules, CAS Center for Excellence in Biomacromolecules, Institute of Biophysics, Chinese Academy of Sciences, Beijing 100101, China; Shanghai Clinical Research and Trial Center, Shanghai 201210, China; Shanghai Institute for Advanced Immunochemical Studies and School of Life Science and Technology, ShanghaiTech University, Shanghai 201210, China; Shanghai Clinical Research and Trial Center, Shanghai 201210, China

**Keywords:** ABC exporter-like importer, iron-loaded siderophore, IrtAB, *Mycobacterium tuberculosis*, ABC transporter

## Abstract

The adenosine 5ʹ-triphosphate (ATP)-binding cassette (ABC) transporter, IrtAB, plays a vital role in the replication and viability of *Mycobacterium tuberculosis* (*Mtb*), where its function is to import iron-loaded siderophores. Unusually, it adopts the canonical type IV exporter fold. Herein, we report the structure of unliganded *Mtb* IrtAB and its structure in complex with ATP, ADP, or ATP analogue (AMP-PNP) at resolutions ranging from 2.8 to 3.5 Å. The structure of IrtAB bound ATP-Mg^2+^ shows a “head-to-tail” dimer of nucleotide-binding domains (NBDs), a closed amphipathic cavity within the transmembrane domains (TMDs), and a metal ion liganded to three histidine residues of IrtA in the cavity. Cryo-electron microscopy (Cryo-EM) structures and ATP hydrolysis assays show that the NBD of IrtA has a higher affinity for nucleotides and increased ATPase activity compared with IrtB. Moreover, the metal ion located in the TM region of IrtA is critical for the stabilization of the conformation of IrtAB during the transport cycle. This study provides a structural basis to explain the ATP-driven conformational changes that occur in IrtAB.

## Introduction

Iron, as an enzyme cofactor, is an essential nutrient required in almost all kingdoms of life including the human pathogen, *Mycobacterium tuberculosis* (*Mtb*) (De Voss et al., 2000; Rodriguez and Smith, 2006; Ryndak et al., 2010). However, for *Mtb*, the amount of free iron is severely limited by resources in the human host (Skaar, 2010; Begg, 2019), since it is mainly stored in metal-binding proteins such as ferritin (Arosio et al., 2017), lactoferrin (Brock, 2012), transferrin (de Jong et al., 1990) and intracellular heme (Paoli et al., 2002). In order to scavenge iron from the human host and in turn to promote infection and pathogenicity (De Voss et al., 2000; Wells et al., 2013), *Mtb* possesses two classes of high-affinity iron siderophores (Snow and White, 1969; Gobin et al., 1995). These are lipid-bound mycobactin (MBT) and a secreted water-soluble variant, carboxymycobactin (cMBT) (Snow and White, 1969; Gobin et al., 1995).

The heterodimeric complex IrtAB has been identified as being responsible for the import of the two iron-loaded siderophores (Fe^3+^-MBT and Fe^3+^-cMBT) from the periplasmic space into the cytoplasm of *Mtb* and other mycobacteria (Rodriguez and Smith, 2006; Arnold et al., 2020). This protein complex is vital for the replication and viability of *Mtb* in macrophages, which reside in the lung alveoli (Rodriguez and Smith, 2006; Ryndak et al., 2010). IrtAB belongs to the adenosine 5ʹ-triphosphate (ATP)-binding cassette (ABC) transporter superfamily (Braibant et al., 2000). Its function of importing iron-loaded siderophores is driven by the binding and hydrolysis of ATP (Rodriguez and Smith, 2006; Arnold et al., 2020). Like other ABC transporters, IrtAB contains two transmembrane domains (TMDs) and two nucleotide-binding domains (NBDs), with a single TMD and a single NBD in each of IrtA and IrtB (Braibant et al., 2000). A unique feature of IrtAB is that IrtA contains an N-terminal siderophore interaction domain (SID) that controls the reduction of Fe^3+^-MBT by an electron donor, flavin-adenine dinucleotide (FAD), and this domain is essential for the uptake of Fe^3+^-MBT (Ryndak et al., 2010; Arnold et al., 2020). In contrast, the SID of IrtA is not essential for the acquisition of Fe^3+^-cMBT (Arnold et al., 2020). Interestingly, although IrtAB functions as an ABC importer, it adopts the canonical fold of type IV ABC exporters (Thomas et al., 2020), which are normally responsible for the efflux of intracellular substrates such as the multidrug resistance protein, Sav1866 (Dawson and Locher, 2006). In addition, IrtAB does not require a substrate-binding protein (SBP) to capture specific substrates in the periplasmic space (Arnold et al., 2020), whereas canonical ABC importers such as maltose transporter, MBP-MalFGK_2_ (Oldham et al., 2007), and trehalose transporter, LpqY-SugABC (Liu et al., 2020), require this assistance.

Based on multiple crystal and cryo-electron microscopy (cryo-EM) structures, the transport mechanism of canonical ABC exporters and importers is well understood (Chen, 2013; Rice et al., 2014; Locher, 2016; Mi et al., 2017; Hofmann et al., 2019). Despite the fact that structures for three transporters, YbtPQ (Wang et al., 2020), Rv1819c (Rempel et al., 2020) and ABCD4 (Xu et al., 2019), have been determined, the precise details as to how these ABC exporter-like importers, especially importers with a type IV exporter fold, operate have remained elusive. To date, there is also limited information to explain how IrtAB mediates the import of iron-loaded siderophores across the membrane in *Mtb*. This is highlighted by the fact that, amongst all mycobacteria, only the crystal structure of SID-truncated IrtAB and the low-resolution (6.9 Å) cryo-EM structure of full-length IrtAB have been determined, in the nucleotide-free state (Arnold et al., 2020). Both structures were determined for the *Mycobacterium thermoresistibile* version of the protein, which shares 71% sequence identity with its *Mtb* counterpart. Therefore, in order to fully understand the complete functionality of IrtAB, more structures in differently liganded states are required.

Herein, we have used cryo-EM to determine the structure of unliganded *Mtb* IrtAB and its structure in complex with ATP, ADP, or the ATP analogue (AMP-PNP). These structures provide valuable insights into the molecular basis for the acquisition of iron-bound siderophores by pathogens.

## Results

### Biochemical characterization

**Figure 1.**
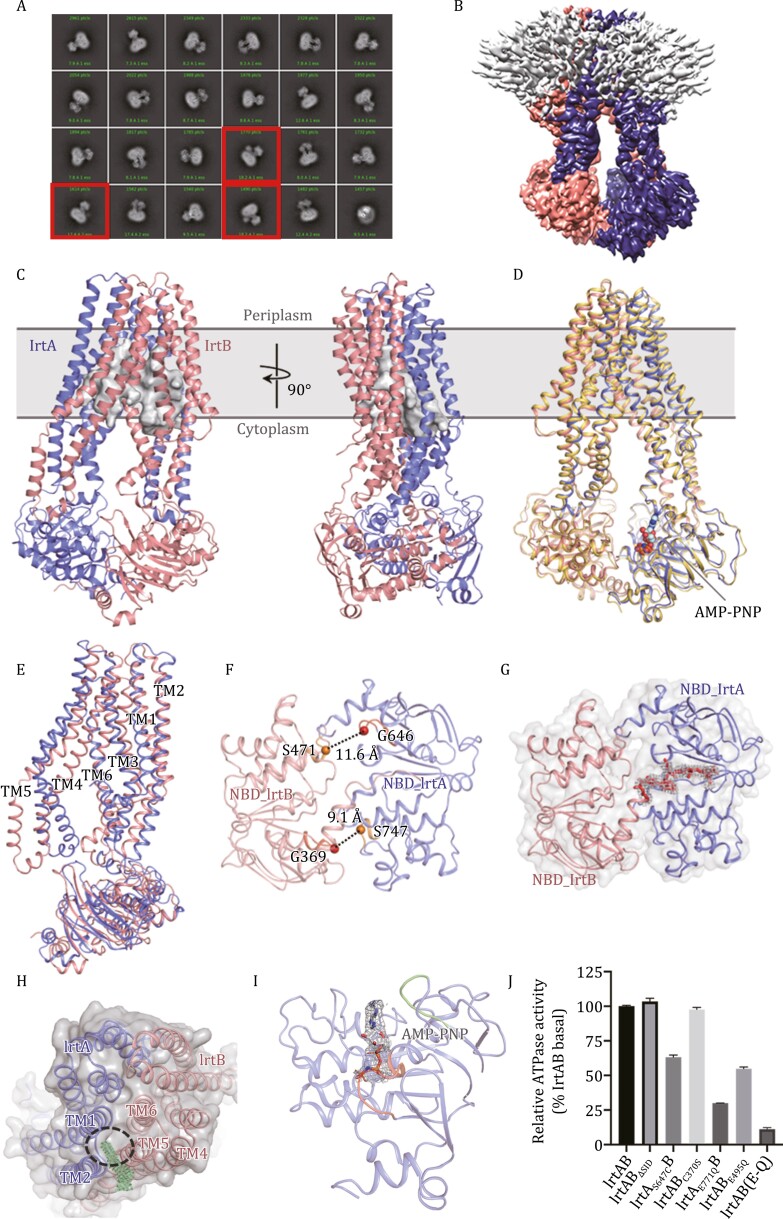
**Cryo-EM structures of IrtAB from *Mtb*.** (A) Representative 2D classification averages showing full-length IrtAB in different orientations. The SID of IrtA is observed in some views indicated by the red boxes. (B) Cryo-EM map of full-length IrtAB. IrtA is in slate, IrtB is in salmon, the detergent micelle is in grey. (C) Cartoon representation of inward-facing IrtAB viewed from the plane of the membrane. The membrane is shown in grey. IrtA and IrtB are in slate and salmon, respectively. The cavity of IrtAB is shown as a grey surface. (D) Superposition of ATP-free (yellow) and one AMP-PNP-bound (IrtA, slate; IrtB, salmon) structures. The ATP analogue AMP-PNP is drawn as spheres and binds to the NBD of IrtA. (E) Superimposition of IrtA (slate) and IrtB (salmon) structures. The transmembrane helices are numbered. (F) The asymmetric opening of the NBD interface. The distances between equivalent residues in the two ATPase sites are indicated. The Walker A and the ABC signature motifs in NBDs are coloured red and orange, respectively. (G) View of the NBDs of IrtAB without nucleotide bound from the cytoplasm. The NBDs are shown as transparent surface and in cartoon representation. The density map of C-terminal extension of the NBD of IrtB (red cartoon) is shown as grey mesh and contoured at 10 σ. (H) View of the TM region interface between IrtA and IrtB. Non-protein cryo-EM map density contoured at 7 σ is shown as green mesh. (I) Cartoon representation of NBD of IrtA binding AMP-PNP. The Walker A and A-loop are coloured red and green, respectively. The density map of AMP-PNP is shown as grey mesh and contoured at 9 σ. (J) The ATPase activities of five IrtAB variants and SID-truncated IrtAB (IrtAB_ΔSID_) were normalized relative to that of the wild-type (WT) IrtAB. Error bars represent mean ± SD based on three independent measurements.

Full-length *Mtb* IrtAB was overexpressed in *Mycobacterium smegmatis* (*M. smegmatis*) (strain mc^2^ 155), purified in lauryl maltose neopentyl glycol (LMNG) and exchanged into digitonin detergent (Fig. S1A). Two-dimensional (2D) class averages of full-length IrtAB cryo-EM particle images showed clear features with the SID of IrtA clearly visible in some 2D class orientations (Figs. 1A and S2). However, there was no SID observed in the final 3D reconstruction of the unliganded protein (Figs. 1B and S2). It is suspected that this may be related to the long flexible loop at the join between the SID and the first elbow helix of the transmembrane (TM) region of IrtA (Fig. S3A). This flexible link could cause the SID of IrtA to swing relative to the TM region. Considering that the SID of IrtAB is not essential for the transport of Fe^3+^-cMBT across the membrane in mycobacteria (Arnold et al., 2020), and that the random swinging of SID could influence structural studies, we expressed and purified SID-truncated IrtAB (IrtAB_ΔSID_) (Fig. S1C) and showed that the ATPase activity of IrtAB_ΔSID_ is comparable to that of the wild-type (WT) protein (Fig. 1J). This suggests that the SID of IrtA does not allosterically regulate ATP hydrolysis of NBDs.

### The cryo-EM structures of *Mtb* IrtAB, inward-facing states

The cryo-EM structure of full-length IrtAB without a bound nucleotide resembles the canonical fold of type IV ABC exporters (Perez et al., 2015; Mi et al., 2017; Thomas et al., 2020), but its TM region has a partially collapsed inward-facing configuration (Fig. 1C). The two polypeptide chains of IrtAB are asymmetric (RMSD of 4.37 Å after superimposition of 336 Cα atoms) in this state (Fig. 1E), with the largest difference located in the TMDs of IrtA and IrtB with an RMSD of 7.15 Å for 139 aligned Cα atoms, especially in the TMHs 4–6. Moreover, the opening of the NBD interfaces of IrtAB also shows an asymmetric feature where the Walker A and signature motifs located near the catalytic site of NBD in IrtB are closer to each other than those of the catalytic site of NBD in IrtA (Fig. 1F), similar to that of the cystic fibrosis transmembrane conductance regulator (CFTR), an ATP-gated chloride channel (Zhang and Chen, 2016), but this asymmetry appears to be caused by the interactions between the C-terminal extension of IrtB NBD and the NBD of IrtA (Fig. 1G).

The overall fold of *Mtb* IrtAB is similar to that of the crystal structure of the SID-truncated counterpart from *Mycobacterium thermoresistibile* (Arnold et al., 2020) (RMSD of 2.38 Å after superimposition of 1,021 Cα atoms) (Fig. S3B). However, there are several differences between these two structures, which may be, in part, due to the different methods used for structure determination. For the IrtA subunit, the differences are mainly located in the TM region, where the transmembrane helices (TMHs) have different degrees of shift at the cytoplasmic and periplasmic sides with TMH4 (flanking helix) and TMH5 having the largest conformational changes (Fig. S3C). Moreover, TMH3 also shows an increased displacement at the cytoplasmic side. By contrast, the TM region and NBD of IrtB are both different in these two structures (Fig. S3D). The changes to the TM region in IrtB are similar to that observed in IrtA. Moreover, TMH5 is broken at the cytoplasmic side in the cryo-EM structure. The NBDs of IrtB show a significant translational shift, which could be related to the binding of the antibody at this position in the crystal structure. Furthermore, in the cryo-EM map, a strong continuous non-protein density was found at the interface of IrtA and IrtB at the periplasmic side (Fig. 1H). Specifically, this is between TMH2 in IrtA and TMH5 in IrtB. This density appears to belong to a partially ordered lipid molecule, implying that such molecules may be involved in stabilizing the complex.

We next determined the structure of IrtAB_ΔSID_ at 2.9 Å resolution in the presence of 1 mmol/L AMP-PNP-Mg^2+^, adenosine 5'-(β, γ-imido) triphosphate, an inert ATP analogue (Figs. 1D and S4). Surprisingly, IrtAB still adopts an inward-facing conformation similar to the nucleotide-free full-length structure (Fig. 1D). The density for AMP-PNP is observed near the A-loop and Walker A motif of the NBD only in IrtA (Fig. 1I). AMP-PNP is not visible in IrtB. Superimposition of full-length IrtAB and this structure (IrtAB_ΔSID_-AMP-PNP bound) gives an RMSD of 0.85 Å for 1,074 Cα atoms, which suggests the SID of IrtAB has little effect on the structures of the TM regions and NBDs for this transporter, consistent with the fact that SID has no effect on the ATPase activity of IrtAB (Fig. 1J).

The structure of this complex further shows that there is an asymmetric binding of the nucleotides in IrtAB, and implies that the NBDs of IrtA and IrtB have different binding affinities for nucleotides. To test this observation, we increased the concentration of nucleotides when preparing cryo-EM samples and determined the structure of IrtAB_ΔSID_ in the presence of 10 mmol/L AMP-PNP-Mg^2+^ (Fig. S5). As expected, two AMP-PNP molecules were visible in the NBDs of IrtAB, even though IrtAB_ΔSID_ still exhibits an inward-facing conformation (Fig. S5H). Different binding preferences of NBDs to nucleotides have also been observed in the heterodimeric ABC exporter, TM287/288 (Hohl et al., 2012). However, in that case, the NBDs contain one degenerate site and one consensus site (Procko et al., 2009; Hohl et al., 2012). It has also been shown that sequence variations between the second and third glycine residues of the Walker A motifs in NBDs can strongly affect the binding/hydrolysis of ATP in the multidrug resistance-associated protein (MRP1) (Yang et al., 2003). Comparing the sequences of NBDs in IrtA and IrtB shows that Ser647 of the Walker A motif in IrtA is replaced by Cys370 at the corresponding position in IrtB (Fig. S10A). This suggests that IrtA and IrtB may have different properties with regard to ATP hydrolysis, as is also observed in MRP1. To test this hypothesis, we interchanged these two residues in the two NBDs of IrtAB and measured the ATPase activities of these two mutants (IrtA_S647C_B and IrtAB_C370S_). The results showed that the S647C mutation reduced ATP hydrolysis of IrtAB by ~40% compared with the WT protein, whereas the C370S mutation in IrtB had almost no effect on ATPase activity (Fig. 1J). Moreover, the inactivation of ATPase activity in IrtA has a greater impact on the complex compared with IrtB (Fig. 1J). Taken together, these results show that the NBD of IrtA has a higher affinity for nucleotides and increased ATPase activity than that of IrtB.

In addition, we also solved the structure of IrtAB_ΔSID_ in complex with ADP (Fig. S6 and Table S1). As expected, this structure adopts a similar conformation to that of the ATP-free state (RMSD of 0.75 Å after superimposition of 1035 Cα atoms). Two obvious ADP densities are present in the NBDs of IrtA and IrtB (Fig. S6H). More likely, this structure represents the post-hydrolytic state, where ADP has not yet been released.

### Structure of an IrtAB mutant in complex with ATP has an occluded state

**Figure 2.**
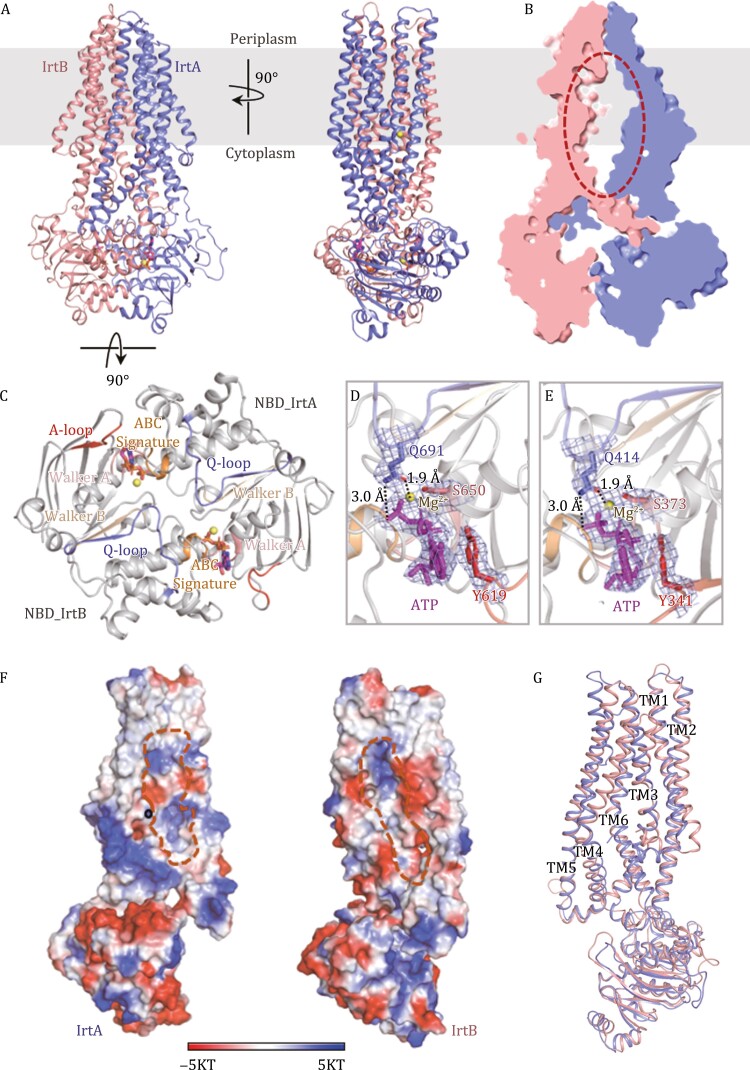
**Cryo-EM structure of IrtAB**_**ΔSID**_ **(E-Q) in complex with ATP.** (A) Two orthogonal views of the ATP-bound IrtAB_ΔSID_ (E-Q). ATP is represented as a stick model coloured magenta, and Mg^2+^ ions are shown as yellow spheres. (B) Slice-through representation of the internal cavity of IrtAB. The amphipathic cavity is indicated by a dashed red ellipse. (C) View of the NBD dimer from the periplasm. The highly conserved sequence motifs are also indicated. (D) Molecular interactions at the IrtA ATPase site, together with the density map (mesh) for ATP-Mg^2+^ and nearby residues. Conserved key residues are drawn as stick models. Interactions between glutamine in the Q-loop and ATP or Mg^2+^ are indicated by dashed lines. (E) Molecular interactions at the IrtB ATPase site, together with the density map (mesh) for ATP-Mg^2+^ and nearby amino acids. Conserved key residues are drawn as stick models. Interactions between glutamine in the Q-loop and ATP or Mg^2+^ are indicated by dashed lines. (F) The electrostatic inner surface representation of IrtA and IrtB in the occluded conformation. The cavity is outlined with a broken orange line. The surfaces are colored from blue (most positive) to red (most negative). Hydrophobic regions are shown in white. The binding site of the metal ion in IrtA is indicated with a black circle. (G) Superimposition of IrtA (slate) and IrtB (salmon) structures.

To trap IrtAB in its ATP-bound state, the catalytic glutamate residues (Glu771 in IrtA and Glu495 in IrtB) were replaced by glutamine (IrtAB (E-Q)). We firstly tried to determine the structure of full-length IrtAB (E-Q) in complex with ATP, but this sample was unstable under freezing conditions. By contrast, the SID-truncated IrtAB (E-Q) (IrtAB_ΔSID_ (E-Q)) incubated with 10 mmol/L ATP-Mg^2+^ was suitable for cryo-EM and could be resolved at 3.1 Å resolution (Figs. 2A and S7; Table S1). Addition of ATP-Mg^2+^ resulted in an overall conformation that is substantially different from that of the ATP-free form.

At the cytoplasmic side, the two NBDs of IrtA and IrtB form a “head-to-tail” dimer, characteristic of ABC transporters (Fig. 2A and 2C). Two ATP molecules are bound at the dimer interface, each of which is stabilized by an interaction between a Walker A motif in one NBD (IrtA or IrtB) and an ABC signature motif in the other NBD (IrtB or IrtA) (Fig. 2C). Two magnesium ions are also observed, which ligand between the β- and γ-phosphates of ATP, a serine in the Walker A motif and the Q-loop glutamine, near the ABC signature motif (Fig. 2D and 2E). The structures of the two NBDs of IrtAB are similar (RMSD of 1.10 Å after superimposition of 194 Cα atoms), with the differences mainly located in the α-helical subdomains, which are unique to ABC transporters and generally are structurally more diverse than the other subdomains (ter Beek et al., 2014). The interactions between the two ATP molecules and nearby residues are very similar in IrtAB (Fig. S8). The density for the C-terminal extension of the NBD of IrtB is not observed, thus this region appears to undergo destabilization upon ATP binding.

The TMHs of IrtAB do not split into two wings when ATP binds (Fig. 2A). As a result, the TMHs pack closely together to form an occluded amphipathic cavity with an approximate volume of ~4,600 Å^3^ (~2,700 Å^3^ in IrtA and ~1,900 Å^3^ in IrtB) (Fig. 2B and 2F), which spans the entire thickness of the membrane and extends to the intracellular part of IrtAB. Therefore, the structure determined here represents an intermediate state between the inward- and outward-facing conformation, similar to that observed for the recently published yersiniabactin (a siderophore) importer YbtPQ from uropathogenic *Escherichia coli* (UPEC), where it has an occluded conformation trapped by adenosine 5ʹ-diphosphate (ADP)-Mg^2+^-vanadate complex (Hu et al., 2021).

### A metal ion binding site in the TM region of IrtA

**Figure 3.**
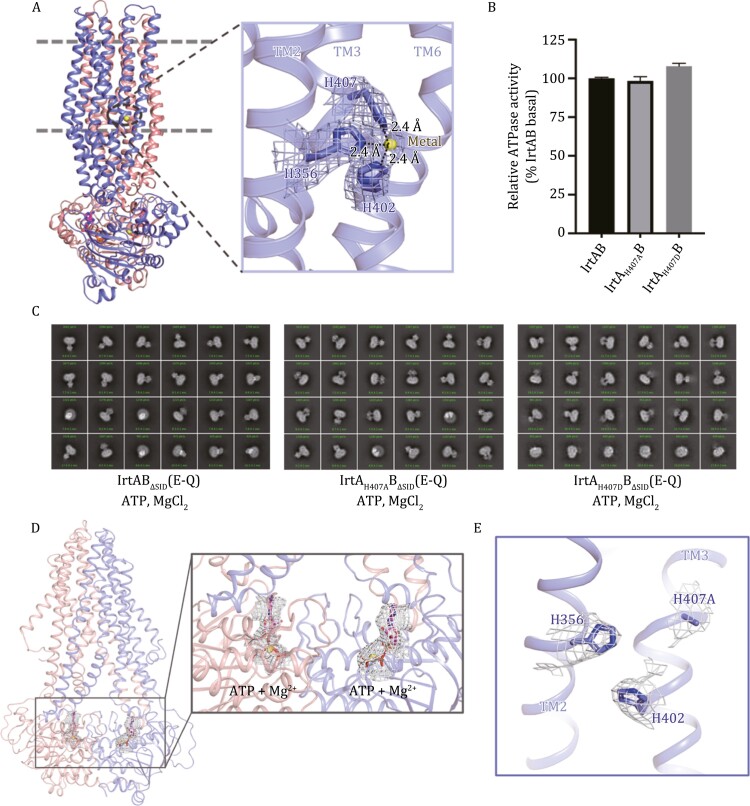
**The metal ion binding site of IrtAB in the occluded state.** (A) Close-up view of the metal ion binding site in the TM region of IrtA. The density map for metal ion and residues that ligand to this metal is shown in mesh. Interactions between histidine and metal ion are indicated by dashed lines. (B) The ATPase activities of two variants in metal ion binding site were normalized relative to that of the wild-type (WT) IrtAB. Error bars represent mean ± SD based on three independent measurements. (C) Representative cryo-EM 2D class averages of IrtAB_ΔSID_ (E-Q) and two variants in the presence of 10 mmol/L ATP and MgCl_2_. (D) Cartoon representation of the structure of IrtA_H407A_B_ΔSID_ (E-Q) in complex with ATP. The density maps for ATP (magenta sticks) and Mg^2+^ (yellow spheres) are shown as grey mesh and contoured at 9 σ. (E) The density map for the metal ion binding site in IrtA_H407A_ is shown as grey mesh and contoured at 7.5 σ.

Like IrtAB in *Mtb*, YbtPQ is responsible for the import of metal-chelated yersiniabactin into UPEC to acquire necessary metal ions such as Fe^3+^, Cu^2+^, and Zn^2+^ (Koh et al., 2015; Wang et al., 2020). The marked difference between these two structures in the occluded state is that an additional unknown metal ion is observed in the cavity of IrtAB (Fig. 3A). To our knowledge, such a metal has not been found in any other ABC transporter. Based on the stoichiometry of binding, this metal is most likely a divalent metal ion such as Ni^2+^, Zn^2+^, or Co^2+^. However, the precise identity of the metal ion needs to be identified with more studies. This metal is bound to IrtA only and is liganded to three histidine residues (His356, His402, and His407) (Fig. 3A), which are highly conserved across mycobacteria. In contrast, one of the histidine (His407) in IrtA is substituted by aspartate at the corresponding position in YbtP (Fig. S10B). To initially investigate the role of this unusual metal ion in IrtAB, we generated two mutants of His407, one to alanine and the other to aspartate like YbtPQ, to disrupt metal ion binding. The ATPase activities of both variants were comparable to that of the WT protein (Fig. 3B). Next, using a similar strategy as obtaining the structure of IrtAB bound ATP, we performed cryo-EM analysis of His407 mutation samples (IrtA_H407A_B_ΔSID_ (E-Q) and IrtA_H407D_B_ΔSID_ (E-Q)) incubated with 10 mmol/L ATP-Mg^2+^. The 2D class averages showed that the NBDs of most molecules in these two samples are separated, a result similar to that of the WT sample without the addition of nucleotides, but is significantly different from IrtAB_ΔSID_ (E-Q) with ATP-Mg^2+^ bound (Fig. 3C). As observed in 2D class averages, the cryo-EM structure of IrtA_H407A_B_ΔSID_ (E-Q) under the condition of ATP-Mg^2+^ resembles the inward-facing conformation of *apo*-IrtAB and no metal ion was observed in the TM region of IrtA (Figs. 3D, 3E and S9). Together, these data suggest that the metal ion in the TM region plays a vital role in stabilizing the conformation of IrtAB during the transport cycle. However, we cannot rule out that it has other functions.

### Conformational changes upon ATP binding

**Figure 4.**
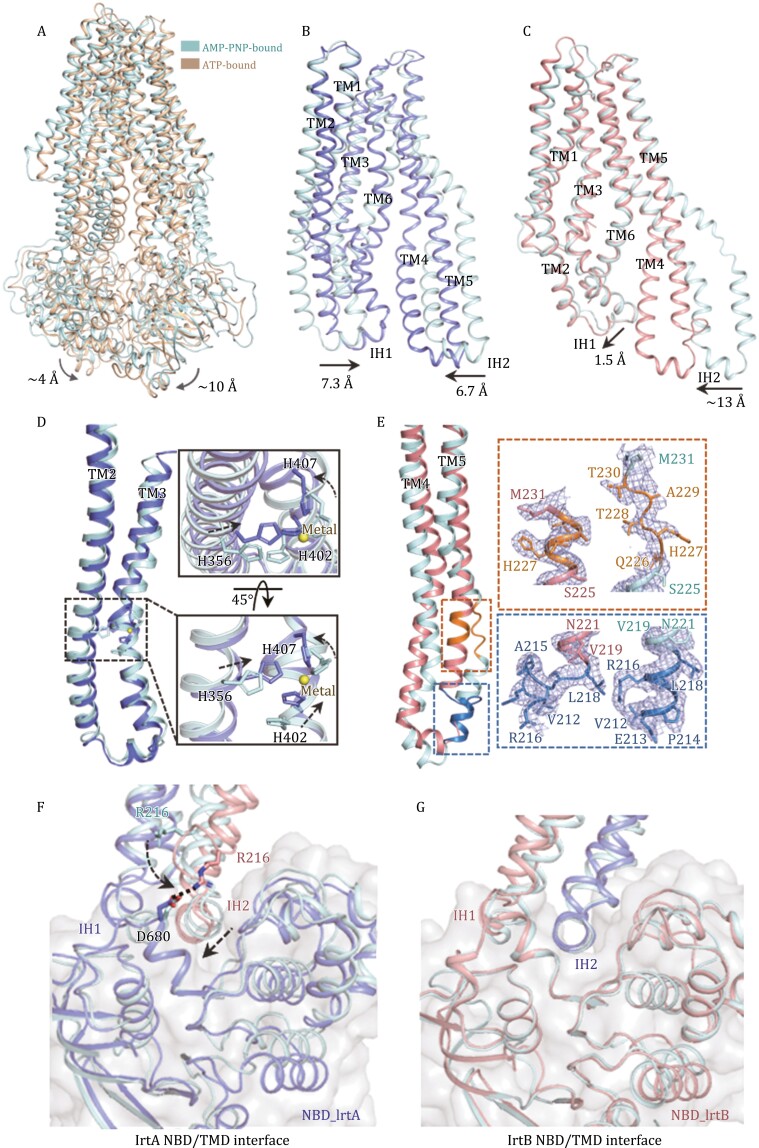
**Conformational changes to IrtAB upon ATP binding.** (A) Superposition of ATP-bound (gold) and AMP-PNP-bound (palecyan) structures. (B) Superposition of the TM region of IrtA in ATP-bound state (slate) and AMP-PNP-bound state (palecyan). The shift of the intracellular helices (IH) is indicated. (C) Superposition of the TM region of IrtB in ATP-bound state (salmon) and AMP-PNP-bound state (palecyan). The shift of the intracellular helices (IH) is indicated. (D) Close-up view of the conformational changes of metal ion binding site in the TM region of IrtA upon ATP binding. (E) The structural rearrangements of TMH5 in IrtB upon ATP binding. (F and G) Cartoon representation of the NBD/TMD interface in IrtA (F) and IrtB (G) before and after ATP binding. Structures of the inward-facing IrtAB (AMP-PNP-bound, palecyan) and the occluded IrtAB (ATP-bound) are superposed with respect to the NBDs. The surface clefts where the intracellular helices are docked into, are outlined in grey. The shift of IH2 in IrtB and the conformational change of Arg216 in TMH5 of IrtB are also indicated by the dashed arrows. Interaction between Arg216 in IrtB and Asp680 in IrtA is indicated by the dashed line.

Upon ATP binding, IrtA and IrtB rotate towards the centre of the IrtAB complex, but these conformational changes are asymmetric with the largest displacement observed at the C-terminal ends of the NBDs of IrtA and IrtB, being ~10 and ~4 Å, respectively (Fig. 4A), which is consistent with the conformational changes to the coupling helix (IH2) in IrtB and IrtA (Fig. 4B and 4C). Interestingly, the conformational changes induced by the addition of ATP-Mg^2+^ make IrtA closely resemble IrtB with an RMSD of 2.59 Å after the superimposition of 309 Cα atoms (Fig. 2G).

The conformational changes of IrtA and IrtB are mainly located in TMHs in the membrane inner leaflet and the NBDs in the cytoplasm, while IrtA has larger changes in comparison with that of IrtB (Fig. 4A). For IrtA, TMHs 2–6 undergo rigid body conformational changes and TMHs 2–3 and TMH6 move towards each other with TMHs 4–5 blocking the lateral opening at the side of IrtA (Fig. 4B). TMHs 4–5 of IrtB undergo a similar conformational change to that of IrtA in order to close the lateral opening at the IrtB side (Fig. 4C). In addition to these global movements, at the binding site of the metal ion in the TM region of IrtA, the three histidine residues (His356, His402, and His407) undergo local conformational changes to optimally coordinate to the metal ion (Fig. 4D). Notably, TMH5 of IrtB undergoes structural rearrangements after ATP binding at the cytoplasmic side (Fig. 4E), which is a unique feature not reported previously in ABC transporters. It is commonly found that TMH4 (flanking helix) adjacent to TMH5 in ABC exporters undergoes structural rearrangements in different transition states in order to control the lateral opening in the membrane inner leaflet (Jin et al., 2012; Kodan et al., 2014; Kim and Chen, 2018). Here, the conformational changes of TMH5 in IrtB may contribute to stabilizing the interaction interface between NBD of IrtA and TMD (NBD_IrtA_–IH2_IrtB_), that is, Arg216 in TMH5 of IrtB interacts with Asp680 of the RecA-like subdomain in the NBD of IrtA by forming a salt bridge (Fig. 4F).

The NBD/TMD interface of the ABC transporter is crucial in coupling the conformational changes during ATP binding and hydrolysis (Kim and Chen, 2018). Multiple crystal structures of the maltose importer, MBP-MalFGK_2_, indicate that the coupling helix rotates relative to the NBDs during the transport cycle (Khare et al., 2009), while studies of P-glycoprotein (P-gp) showed that the NBD and the intracellular helical region of the TMD move as one concerted rigid body in the transport cycle (Esser et al., 2017; Kim and Chen, 2018). In contrast, the two equivalent interfaces in IrtAB (NBD_IrtA_–IH2_IrtB_ and NBD_IrtB_–IH2_IrtA_) show different features during the switch from the inward-facing to occluded conformation (Fig. 4F and 4G), that is, the NBD_IrtA_–IH2_IrtB_ interface is similar to that of the maltose importer, where the IH2_IrtB_ rotates relative to the NBD_IrtA_, whereas the NBD_IrtB_–IH2_IrtA_ interface is largely unchanged, similar to that of P-glycoprotein, where NBD_IrtB_–IH2_IrtA_ moves as one concerted rigid body. Therefore, in the transport cycle, the NBDs and the intracellular helical regions of TMD in IrtAB exhibit an asymmetric allosteric mechanism that is novel in the ABC transporter family.

## Discussion

**Figure 5.**
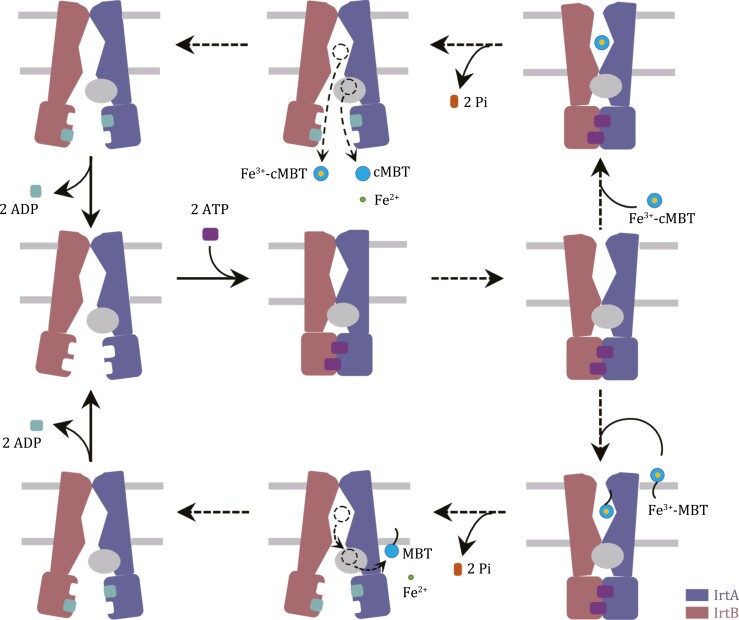
**A putative working transport model.** In the absence of ATP, IrtAB adopts an inward-facing conformation, but has a low-affinity binding pocket. ATP binding is associated with a transition into an occluded conformation, and subsequent formation of a hypothetical nucleotide-bound outward-facing conformation to allow substrates to bind. ATP hydrolysis opens the cytoplasmic gate. Fe^3+^-cMBT could be directly released into the cytoplasm or transferred into the SID of IrtA for the reduction and release of iron. Fe^3+^-MBT could be transferred into SID, while the lipid tail of MBT is embedded in the leaflet of the cytoplasmic side. After ADP release, IrtAB reverts back to the resting state for the next round of transport.

Although the ABC transporter IrtAB plays a vital role in the replication and viability of *Mtb* in human macrophages, we are at an early stage in understanding its molecular mechanism. The structural information presented here provides valuable insights as to how IrtAB carries out its function as an iron-loaded siderophore transporter. We propose an initial plausible working mode of IrtAB (Fig. 5). In the absence of ATP, IrtAB adopts an open-inward conformation, in which, due to TMH6 of IrtB protruding into the central cavity in the TM region, IrtAB represents a low-affinity binding pocket to guarantee unidirectional transport. This is in contrast to typical ABC exporters that generally have a high-affinity binding pocket (Srinivasan et al., 2014; Johnson and Chen, 2017; Alam et al., 2019), which is consistent with their opposite functions. ATP binding causes asymmetric conformational changes to IrtAB, presenting a transition state, which has an occluded conformation. Subsequently, IrtAB forms a hypothetical nucleotide-bound outward-facing conformation, similar to that of the cobalamin transporter ABCD4 bound ATP (Xu et al., 2019). This allows substrates to access the high-affinity binding site in the TM region of IrtAB. ATP hydrolysis opens the cytoplasmic gate. For the substrate, Fe^3+^-MBT, it is transferred from the binding pocket of the TM region into the SID of IrtA to reduce iron in the imported Fe^3+^-MBT for its assimilation, while the lipid tail of MBT is embedded in the leaflet of the cytoplasmic side (Arnold et al., 2020). For water-soluble substrate, Fe^3+^-cMBT, it could be directly released into the cytoplasm or transferred into the SID of IrtA, and the part released into the cytoplasm could be reduced by other ferric reductases (Arnold et al., 2020). After ADP release, IrtAB reverts back to the resting state for the next round. However, many details of the transport cycle of IrtAB, such as an open-outward conformation and how substrate is recruited and released, remain to be elucidated. This could be achieved by further structural and functional studies.

## Materials and methods

### Cloning and expression

The DNA coding sequences of full-length IrtAB (*Rv1348-Rv1349*) and the SID-truncated IrtAB (IrtAB_ΔSID_) were amplified from *Mtb* H37Rv genomic DNA by PCR and cloned into a pMV261 vector, fused with a C-terminal 10× His tag attached to IrtB. All mutations were generated using the Fast Mutagenesis System (TransGen). All constructs were verified by sequencing and then transformed into *M. smegmatis* mc^2^155 cells for expression. The cells were cultivated at 37°C in Luria Broth (LB) liquid media with shaking (220 rpm), supplemented with 50 μg/mL kanamycin, 20 μg/mL carbenicillin and 0.1% (*v*/*v*) Tween 80. The temperature was reduced to 16°C when the OD_600_ reached 1.0 and 0.2% (*w*/*v*) acetamide was added into the cell cultures to induce the overexpression of protein. After 4 days, cells were harvested by centrifugation at 4,000 rpm for 15 min and frozen at −80°C. All constructs were overexpressed using the same protocol as the WT protein.

### Protein purification

Full-length IrtAB, IrtAB_ΔSID_ and mutant proteins were purified by a similar method. Cell pellets were thawed and resuspended in buffer A which contains 20 mmol/L MES, 150 mmol/L NaCl, pH 6.5. The resuspended cells were then lysed by a French Press at 1,100 bar. Cell debris was removed by centrifugation at 12,000 rpm for 15 min at 4°C. The supernatant was collected and ultracentrifuged at 150,000 ×*g* for 1.5 h. The membrane fractions were collected and resuspended in buffer A. After incubating with 0.5% (*w*/*v*) LMNG (Anatrace) for 1.5 h at 4°C, the suspension was centrifuged and the supernatant was loaded onto an Ni-NTA agarose beads (QIAGEN) affinity column supplemented with 10 mmol/L imidazole. The beads were then washed in buffer A supplemented with 50 mmol/L imidazole and 0.005% LMNG, and then exchanged into 0.06% digitonin buffer. The protein was eluted from the beads with buffer A supplemented with 500 mmol/L imidazole and 0.06% digitonin, then concentrated and loaded to a size exclusion chromatography column (Superose 6 Increase 10/300 GL, GE Healthcare) pre-equilibrated with 20 mmol/L MES, 150 mmol/L NaCl, 2 mmol/L DTT, and 0.06% digitonin, pH 6.5. The peak fractions were pooled and concentrated for cryo-EM sample preparation using a 100-kDa cut-off concentrator (Merck Millipore). The results of protein purification are shown in Fig. S1.

### ATPase activity assay

The ATPase Activity Assay Kit (Sigma-Aldrich, MAK-113) was used to measure ATPase activity. The protein was diluted to a final concentration of 2 μmol/L, incubated with 1 mmol/L ATP in 40 μL reaction mixture which contained 20 mmol/L MES pH 6.5, 150 mmol/L NaCl, 0.06% digitonin, 5 mmol/L MgCl_2_ for 30 min at 37°C. Next, the enzyme reactions were stopped and the colourimetric product was generated by adding 200 μL of Reagent (MAK-113) into each reaction well. After incubating for an additional 30 min at room temperature, the absorbance at 620 nm in each well was measured. ATPase activity was represented as the amount of phosphate produced from the ATP catalytic reaction. The experiments were performed in triplicate. Results were analysed in GraphPad Prism 5.0.

### Cryo-EM sample preparation

The protein samples were concentrated to 5 mg/mL. For IrtAB_ΔSID_, 1 or 10 mmol/L adenosine 5'-(β, γ-imido) triphosphate (AMP-PNP) and MgCl_2_ or 5 mmol/L ADP-MgCl_2_ and 5 mmol/L DTT were added and incubated on ice for 30 min. For mutated IrtAB (E-Q), IrtAB_ΔSID_ (E-Q), IrtA_H407A_B_ΔSID_ (E-Q) and IrtA_H407D_B_ΔSID_ (E-Q), 10 mmol/L ATP-MgCl_2_ and 5 mmol/L DTT were added and incubated on ice for 30 min. All protein samples were combined with appropriate ligands and then centrifuged before preparation for freezing samples. Three microlitres of protein sample was applied to holey carbon grids (Quantifoil, R 0.6/1 Cu 200 mesh) which were H_2_/O_2_ glow-discharged for 30 s. After blotting for 3 s, the grids were frozen in liquid ethane using an FEI Vitrobot Mark IV at 8°C and 100% humidity, and then transferred into liquid nitrogen.

### Cryo-EM data collection

For the full-length IrtAB, IrtA_H407A_B_ΔSID_ (E-Q) and IrtA_H407D_B_ΔSID_ (E-Q) samples, cryo-EM data were collected on a 300 kV FEI Titan Krios electron microscope with a Gatan K3 Summit direct electron detector at a nominal magnification of 29,000 with a pixel size of 0.82 Å in super-resolution mode. Movies were recorded for 2.4 s in 40 sub-frames with a total dose of 60 e^−^/Å^2^. For IrtAB_ΔSID_ with 1 mmol/L AMP-PNP-Mg^2+^, IrtAB_ΔSID_ with 5 mmol/L ADP-Mg^2+^ and IrtAB_ΔSID_ (E-Q) with 10 mmol/L ATP-Mg^2+^ samples, a Gatan K2 Summit direct electron detector, equipped with a Gatan quantum energy filter, was used to capture movies at EFTEM magnification of 165,000 in super-resolution mode with a pixel size of 0.82 Å. For IrtAB_ΔSID_ with 10 mmol/L AMP-PNP-Mg^2+^ and IrtAB (E-Q) with 10 mmol/L ATP-Mg^2+^ samples, a Gatan K3 Summit direct electron detector, equipped with a Gatan quantum energy filter, was used to capture movies at EFTEM magnification of 105,000 in super-resolution mode with a pixel size of 0.832 Å.

### Cryo-EM image processing and 3D Reconstruction

For the image processing of full-length IrtAB, a total of 1,875 original movies were generated and a beam-induced correction was performed by MotionCor2 (Zheng et al., 2017), the remaining steps for image processing were performed using cryoSPARC (Punjani et al., 2017). After CTF estimation, poor-quality images with features such as high astigmatism, low CTF fit resolution and large-offset defocus values were manually removed. 428,375 particles were automatically selected and extracted with a box size of 384 pixels from selected images. Several rounds of 2D classification were performed to remove bad particles, and then *Ab-initio* reconstruction was performed with 308,525 particles to generate 3D volumes as templates for heterogeneous refinement, with 143,181 particles converging into one good class. Homogeneous refinement was performed on this convergence class, followed by non-uniform refinement and local refinement. Finally, a density map was obtained with an estimated average resolution of 3.48 Å according to the gold-standard Fourier shell correlation (FSC) cut-off of 0.143 (Grigorieff, 2016). Local resolution ranges were analysed within cryoSPARC. The other datasets were processed in the same way.

### Model building and refinement

The model of full-length IrtAB was manually built, but relied on the 3.48 Å cryo-EM map and the crystal structure of truncated *Mycobacterium thermoresistibile* IrtAB (PDB: 6TEG) (Arnold et al., 2020). This model was docked into the cryo-EM map in UCSF Chimera (Pettersen et al., 2004) and manually adjusted in Coot (Emsley et al., 2010). Several iterations of real-space refinement were performed in PHENIX (Adams et al., 2010). The models for other states of IrtAB complex were generated based on their cryo-EM maps and using the model of full-length IrtAB as a starting point. Nucleotides were fitted as rigid-bodies into the cryo-EM map using Coot. The structures were refined in real space using PHENIX with secondary structure and geometry restraints in place to prevent overfitting. The final atomic model was evaluated using MolProbity (Chen et al., 2010). Cryo-EM data collection and model refinement statistics are shown in Table S1.

All graphics were generated using PyMOL, UCSF ChimeraX (Goddard et al., 2018), UCSF Chimera.

## Supplementary Material

pwac060_suppl_Supplementary_Material

## Data Availability

All data are available in the manuscript. The accession number for the 3D cryo-EM density maps reported in this paper is EMD-32536, EMD-32537, EMD-32538 and EMD-32539. Atomic coordinates for the IrtAB, IrtAB_ΔSID_-AMP-PNP, IrtAB_ΔSID_ (E-Q)-ATP and IrtAB_ΔSID_-ADP structures have been deposited in the Protein Data Bank with identification codes 7WIU, 7WIV, 7WIW and 7WIX.

## Abbreviations

ABC: ATP-binding cassette
ADP: adenosine diphosphate
AMP-PNP: adenosine 5ʹ-(β, γ-imido) triphosphate
ATP: adenosine triphosphate
cMBT: carboxymycobactin
Cryo-EM: cryo-electron microscopy
DTT: dithiothreitol
FSC: fourier shell correlation
IH: intracellular helix
MBT: mycobactin
*Mtb*: *Mycobacterium tuberculosis*
NBDs: nucleotide-binding domains
PCR: polymerase chain reaction
RMSD: root-mean-square deviation
SID: siderophore interaction domain
TM: transmembrane
TMDs: transmembrane domains
TMH: transmembrane helix
UPEC: uropathogenic *Escherichia coli*
WT: wild-type
