# Supplementary material for: Cryo-EM structures for the Mycobacterium tuberculosis iron-loaded siderophore transporter IrtAB
Source: Protein Cell. 2022 Nov 17;14(6):448–58. doi: 10.1093/procel/pwac060 (PMC10246721; doi:10.1093/procel/pwac060)
Supplement: pwac060_suppl_Supplementary_Material [file pwac060_suppl_supplementary_material.pdf]

Supplementary Information for

Cryo-EM structures for the *Mycobacterium tuberculosis* iron-loaded siderophore transporter IrtAB

Shan Sun, Yan Gao, Xiaolin Yang, Xiuna Yang, Tianyu Hu, Jingxi Liang, Zhiqi Xiong, Yuting Ran, Pengxuan Ren, Fang Bai, Luke W. Guddat, Haitao Yang, Zihe Rao, Bing Zhang

Haitao Yang
Email: yanght@shanghaitech.edu.cn

Zihe Rao
Email: raozh@tsinghua.edu.cn

Bing Zhang
Email: zhangbing@shanghaitech.edu.cn

This PDF file includes:

Figures S1 to S10
Table S1

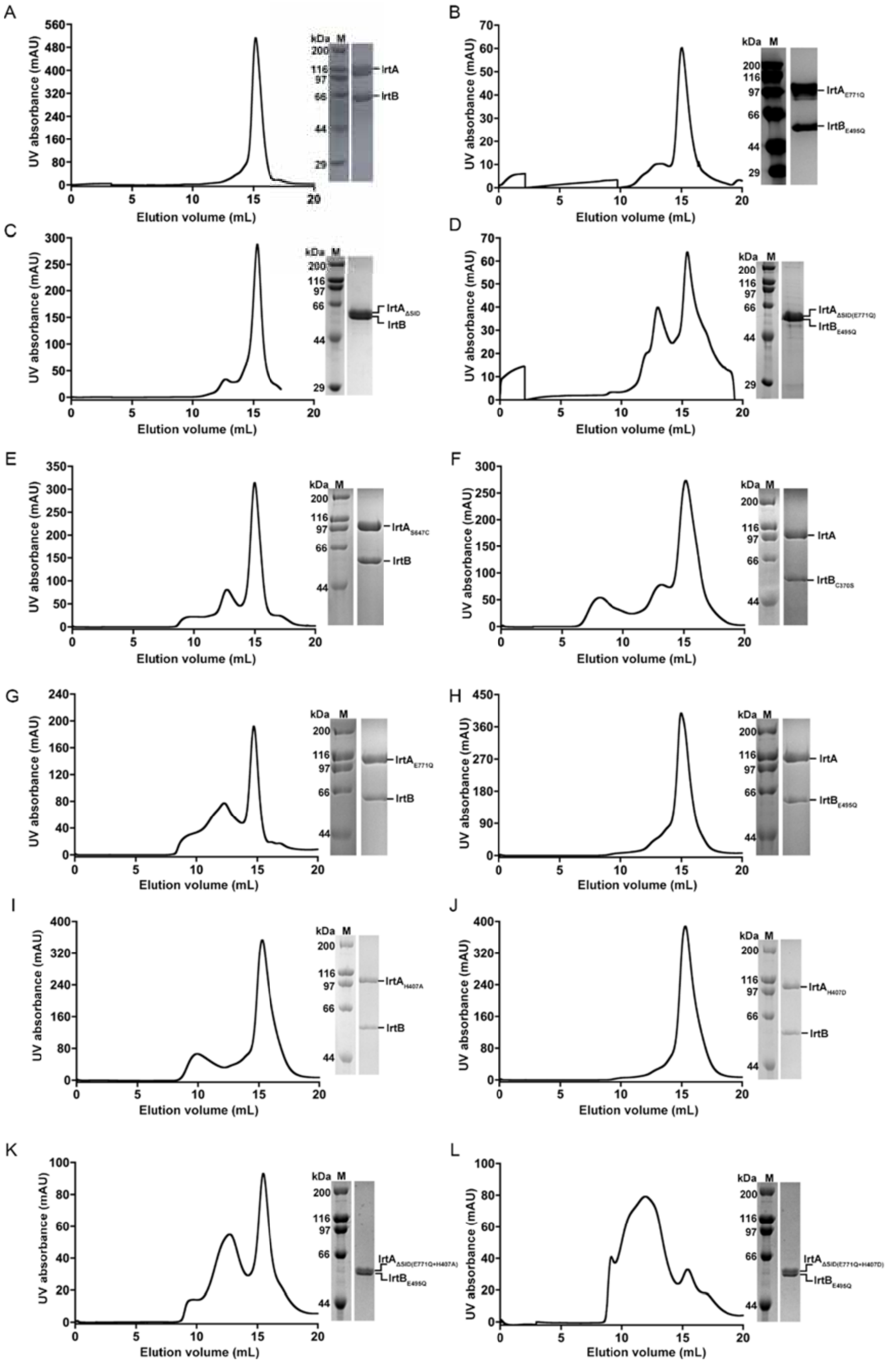

Fig. S1. Properties of *Mtb* IrtAB. (A-L) Size exclusion profile and SDS-PAGE of the purified different samples in digitonin.

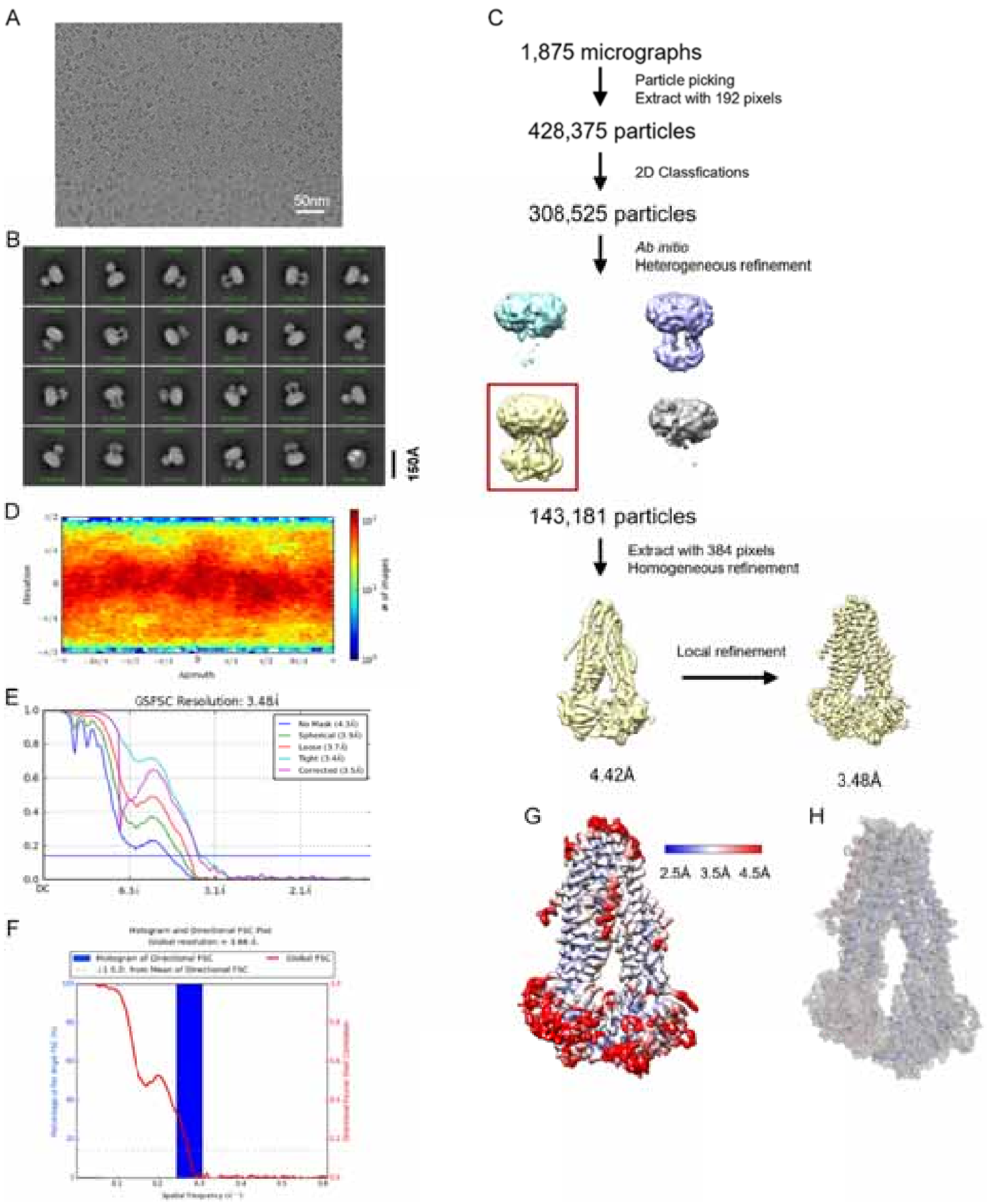

Fig. S2. Cryo-EM data processing of the ATP-free, full-length IrtAB. (A) Representative cryo-EM image of the full-length IrtAB complex. (B) Representative 2D classification averages showing the full-length IrtAB in different orientations. (C) Summary of the image processing procedure. (D) Angular distribution heatmap of particles used for the refinement. (E) Fourier shell correlation (FSC) curves of the final 3D reconstruction. (F) 3D FSC histogram of the final map. (G) Local resolution of the final cryo-EM map of the full-length IrtAB. (H) Cryo-EM map density (gray mesh, contoured at 7 σ) for the structure of full-length IrtAB without nucleotide bound.

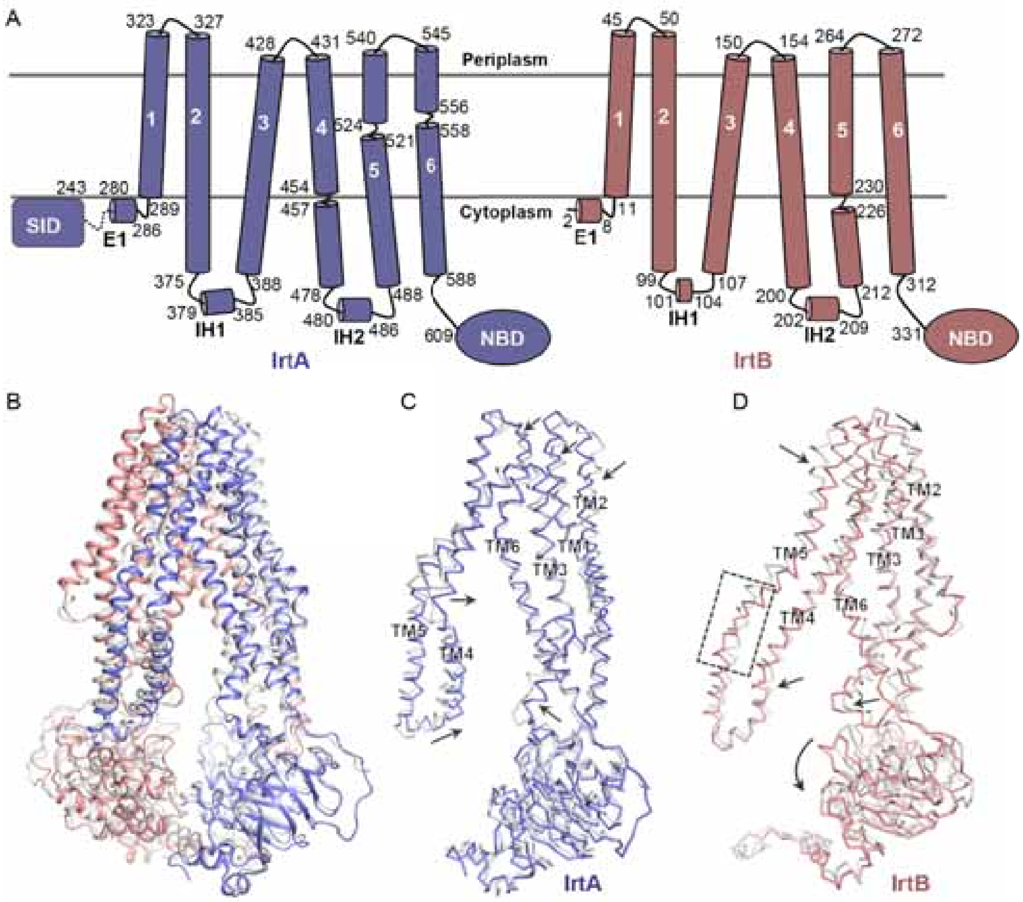

Fig. S3. Structural comparison of IrtAB in mycobacteria. (A) Topology diagram of full-length IrtA (slate) and IrtB (salmon) with TMHs (transmembrane helices) numbered. The amino acid sequence numbers are indicated. The linkage between the siderophore interaction domain (SID) (dotted frame) and elbow helices (E1) is indicated by the dotted lines. IH, intracellular helix. NBD, nucleotide-binding domain. (B) Superposition of *Mtb* IrtAB (IrtA, slate; IrtB, salmon) and *Mycobacterium thermoresistibile* IrtAB (PDB: 6TEJ, white) structures. (C) Superimposition of IrtA structures in *Mtb* (slate) and *Mycobacterium thermoresistibile* (white). The divergence in these two structures is indicated by the black arrows. (D) Superimposition of IrtB structures in *Mtb* (salmon) and *Mycobacterium thermoresistibile* (white). The differences between these two structures are indicated by black arrows and a dashed box.

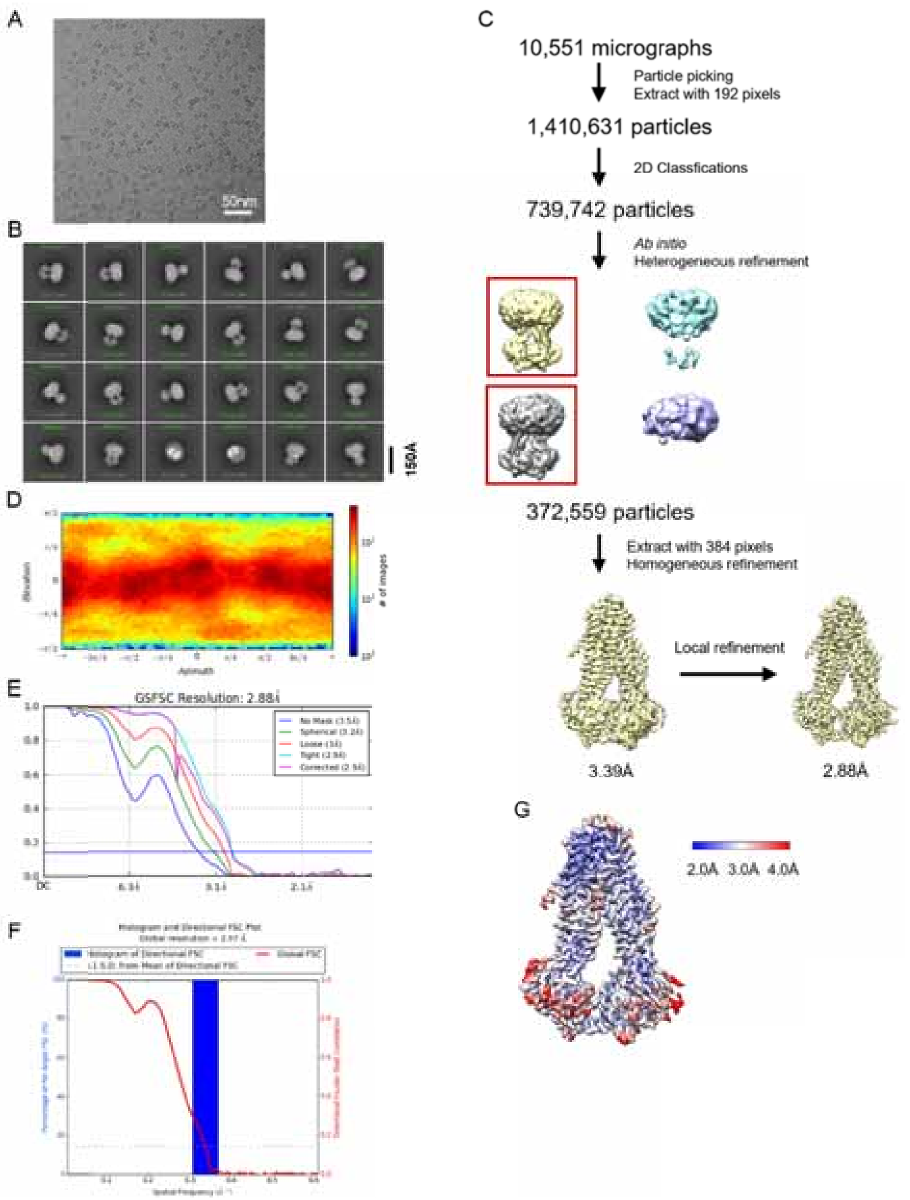

Fig. S4. Cryo-EM data processing of IrtAB$_{\Delta SID}$ under the condition of 1 mM AMP-PNP-Mg^{2+}. (A) Representative cryo-EM image of IrtAB$_{\Delta SID}$ in the presence of 1 mM AMP-PNP-Mg^{2+}. (B) Representative 2D classification averages showing the complex in different orientations. (C) Summary of the image processing procedure. (D) Angular distribution heatmap of particles used for the refinement. (E) Fourier shell correlation (FSC) curves of the final 3D reconstruction. (F) 3D FSC histogram of the final map. (G) Local resolution of the final cryo-EM map of IrtAB$_{\Delta SID}$ in complex with one AMP-PNP molecule.

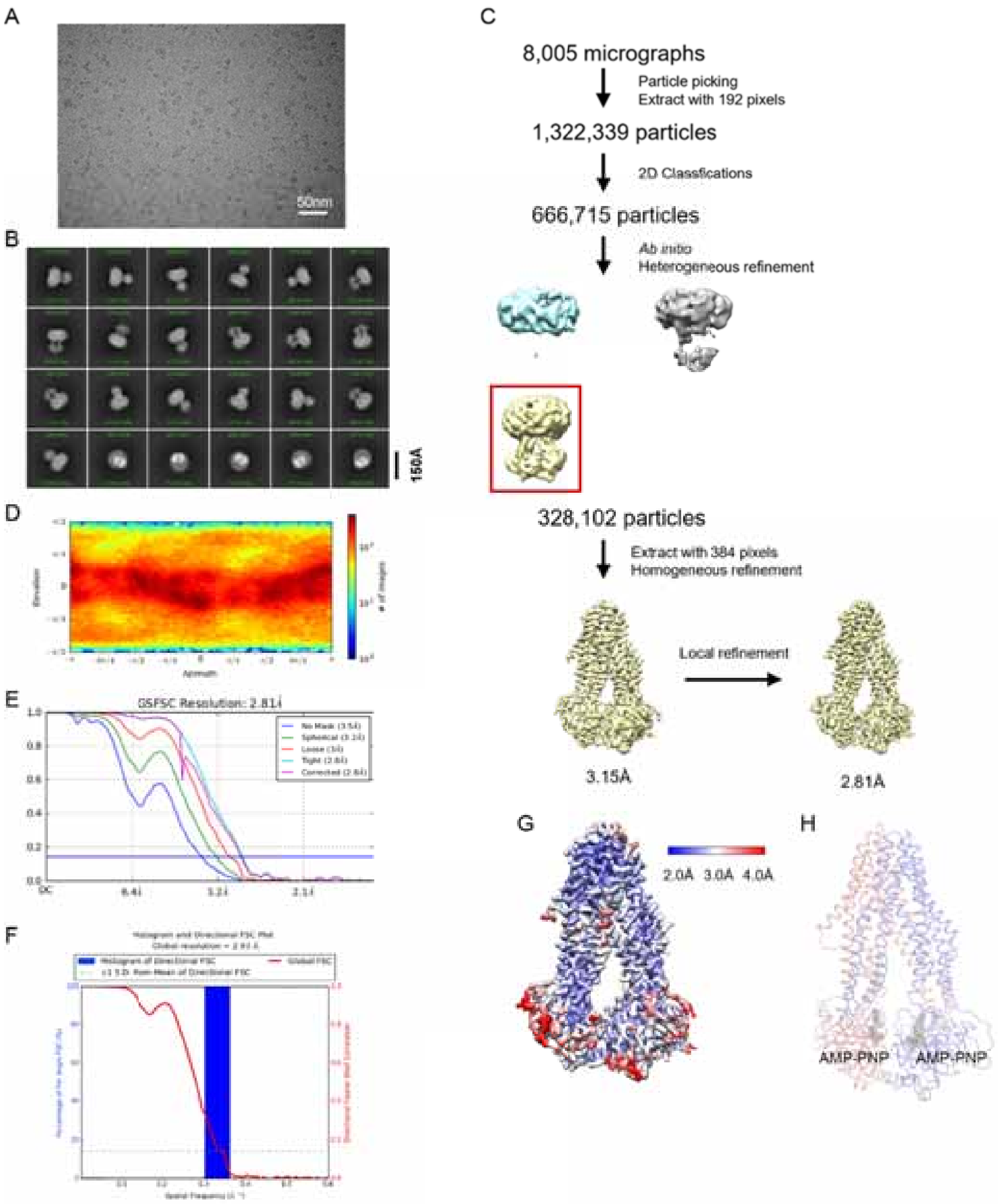

Fig. S5. Cryo-EM data processing of IrtAB$_{\Delta SID}$ under the condition of 10 mM AMP-PNP-Mg^{2+}. (A) Representative cryo-EM image of IrtAB$_{\Delta SID}$ in the presence of 10 mM AMP-PNP-Mg^{2+}. (B) Representative 2D classification averages showing the complex in different orientations. (C) Summary of the image processing procedure. (D) Angular distribution heatmap of particles used for the refinement. (E) Fourier shell correlation (FSC) curves of the final 3D reconstruction. (F) 3D FSC histogram of the final map. (G) Local resolution of the final cryo-EM map of IrtAB$_{\Delta SID}$ in complex with two AMP-PNP molecules. (H) Cartoon representation of the structure of IrtAB$_{\Delta SID}$ in complex with two AMP-PNP molecules. The density maps for AMP-PNP (gray sticks) are shown as gray mesh and contoured at 9 σ.

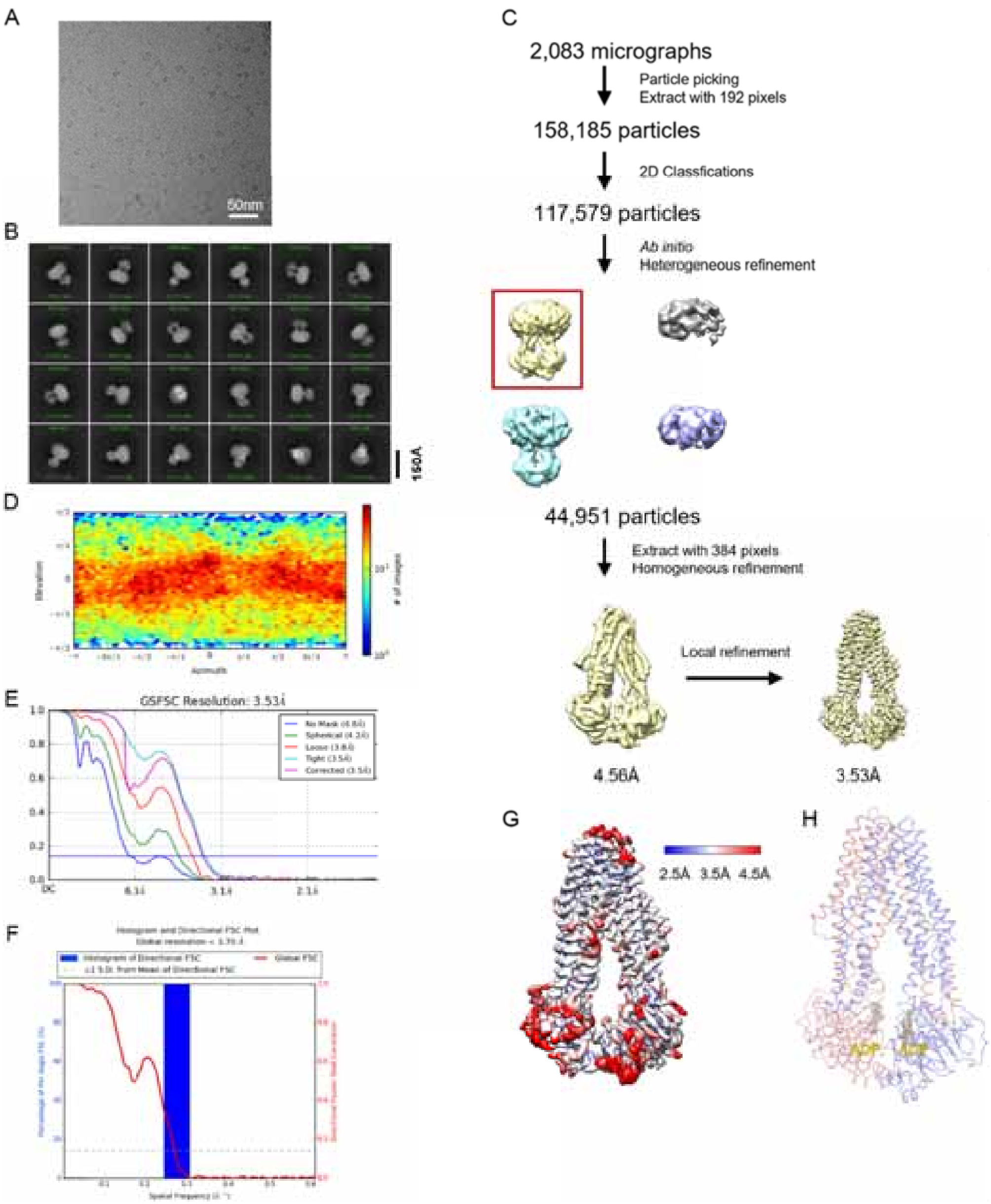

Fig. S6. Cryo-EM data processing of IrtAB$_{\Delta SID}$ with ADP bound. (A) Representative cryo-EM image of IrtAB$_{\Delta SID}$ in complex with ADP. (B) Representative 2D classification averages showing the complex in different orientations. (C) Summary of the image processing procedure. (D) Angular distribution heatmap of particles used for the refinement. (E) Fourier shell correlation (FSC) curves of the final 3D reconstruction. (F) 3D FSC histogram of the final map. (G) Local resolution of the final cryo-EM map of IrtAB$_{\Delta SID}$ in complex with ADP. (H) Cartoon representation of the structure of IrtAB$_{\Delta SID}$ in complex with ADP. The density maps for ADP (yellow sticks) are shown as gray mesh and contoured at 9 σ.

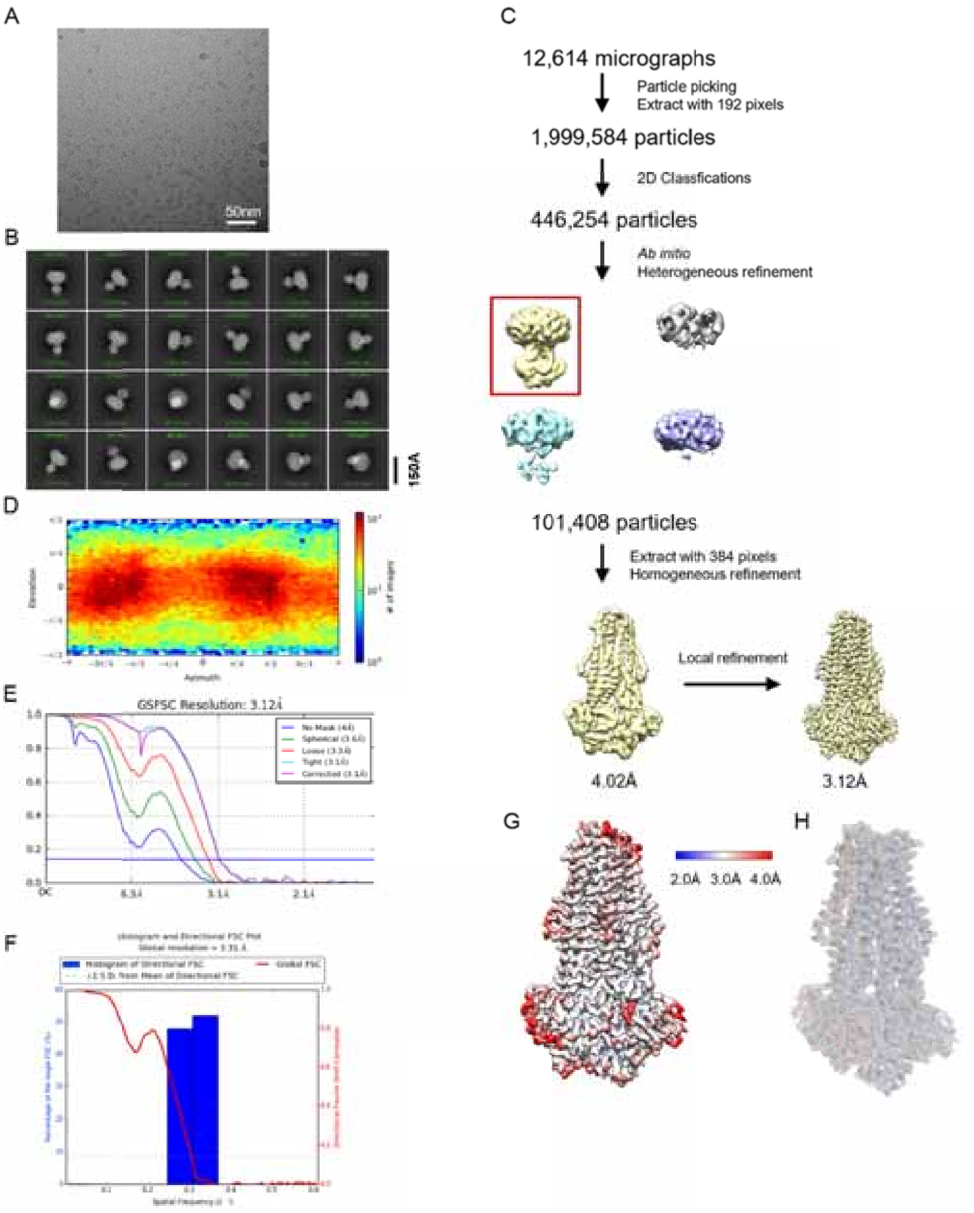

Fig. S7. Cryo-EM data processing of IrtAB$_{\Delta SID}$ (E-Q) bound ATP. (A) Representative cryo-EM image of IrtAB$_{\Delta SID}$ (E-Q) in complex with ATP. (B) Representative 2D classification averages showing the complex in different orientations. (C) Summary of the image processing procedure. (D) Angular distribution heatmap of particles used for the refinement. (E) Fourier shell correlation (FSC) curves of the final 3D reconstruction. (F) 3D FSC histogram of the final map. (G) Local resolution of the final cryo-EM map of IrtAB$_{\Delta SID}$ (E-Q) in complex with ATP. (H) Cryo-EM map density (gray mesh, contoured at 9 σ) for the structure of IrtAB$_{\Delta SID}$ (E-Q) in complex with ATP.

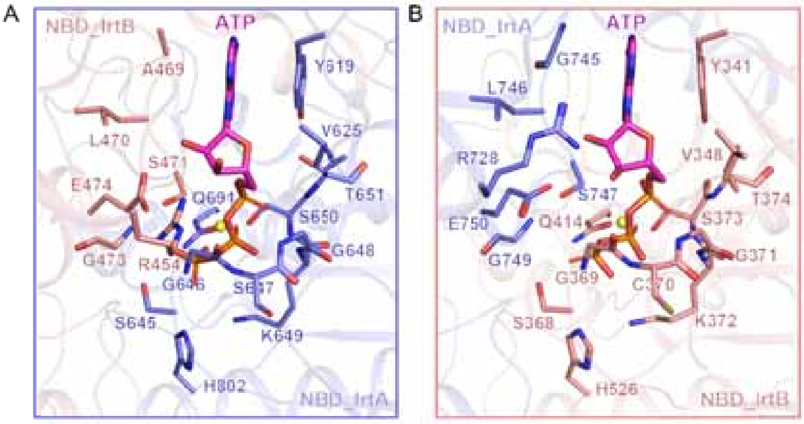

Fig. S8. ATP binding mode of IrtAB in the occluded state. (A) Close-up view of the ATPase site in IrtA. The residues involved in ATP binding are shown as sticks. ATP is represented by magenta sticks colored by heteroatom. The Mg^{2+} is shown as a yellow sphere. (B) Close-up view of the ATPase site in IrtB. The residues involved in ATP binding are shown as sticks. ATP is represented by magenta sticks colored by heteroatom. The Mg^{2+} is shown as a yellow sphere.

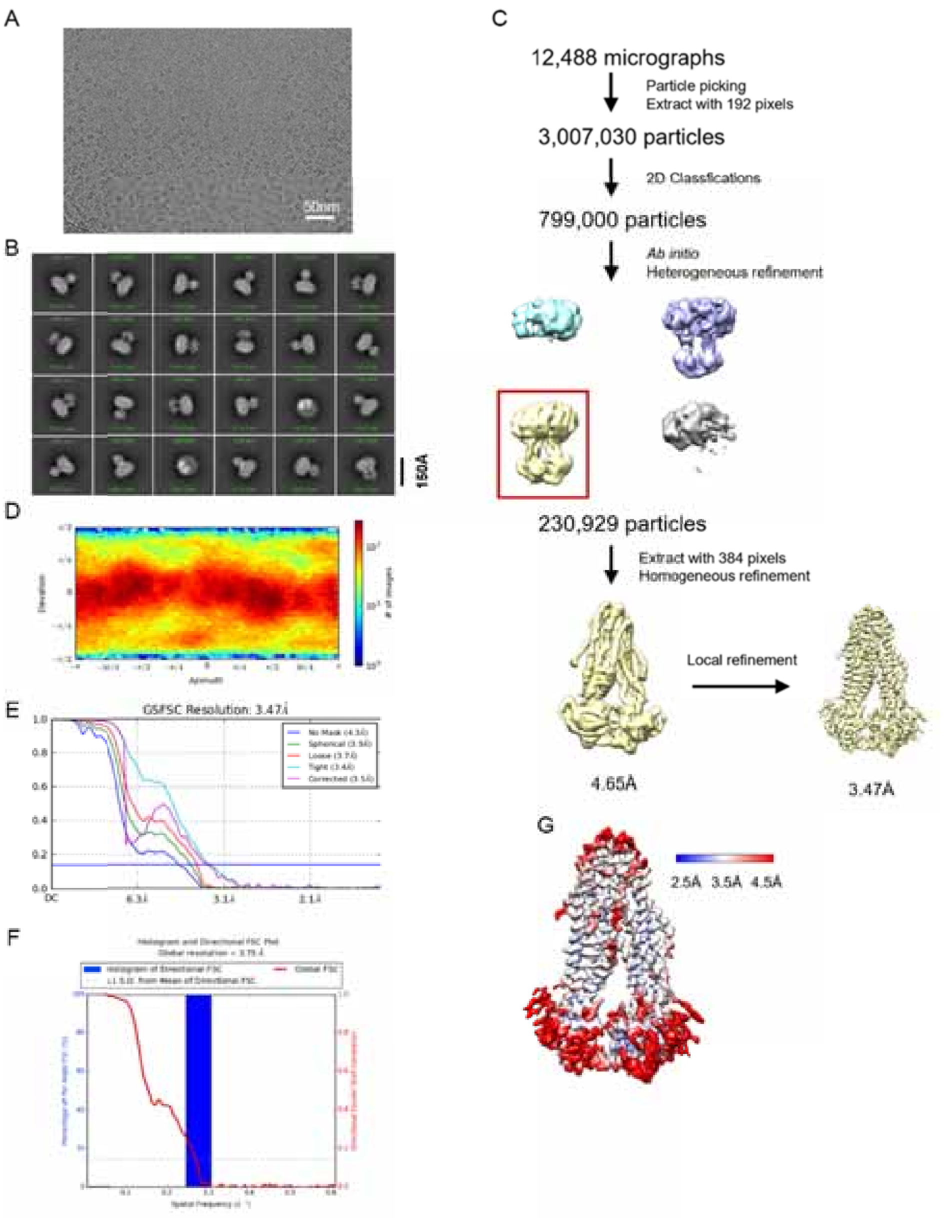

Fig. S9. Cryo-EM data processing of IrtA$_{H407A}$B$_{\Delta SID}$ (E-Q) bound ATP. (A) Representative cryo-EM image of IrtA$_{H407A}$B$_{\Delta SID}$ in complex with ATP. (B) Representative 2D classification averages showing the complex in different orientations. (C) Summary of the image processing procedure. (D) Angular distribution heatmap of particles used for the refinement. (E) Fourier shell correlation (FSC) curves of the final 3D reconstruction. (F) 3D FSC histogram of the final map. (G) Local resolution of the final cryo-EM map of IrtA$_{H407A}$B$_{\Delta SID}$ (E-Q) in complex with ATP.

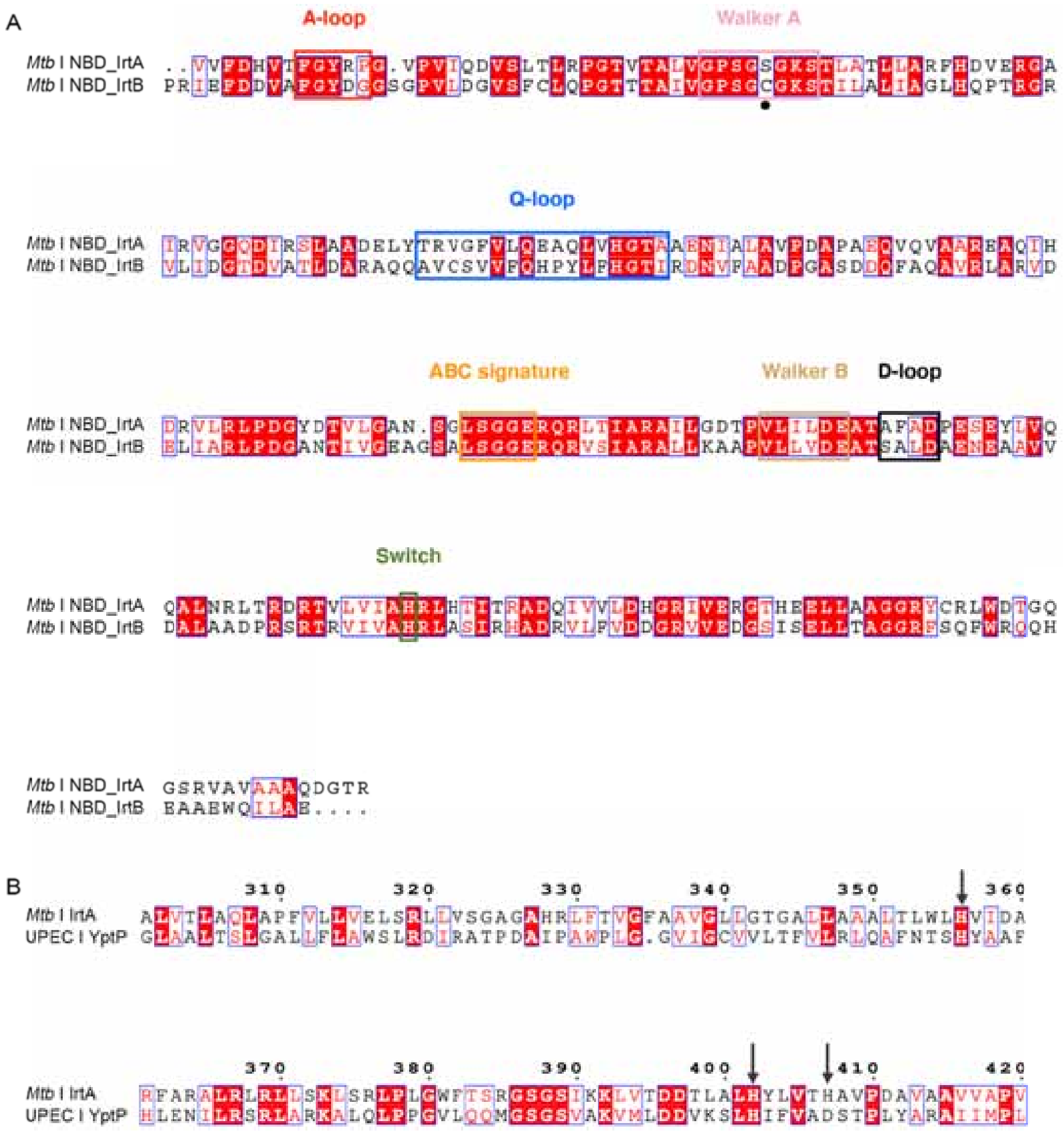

Fig. S10. (A) Sequence alignment of NBDs of IrtA and IrtB. The conserved sequence motifs of NBD are also indicated. (B) The partial sequence alignment of *Mtb* IrtA and uropathogenic *E. coli* (UPEC) YbtP. The residues involved in metal ion binding are marked with black arrows.

Table S1. Statistics for the cyro-EM structures presented in this study.

	IrtAB	IrtAB$_{\Delta SID}$-AMP-PNP	IrtAB$_{\Delta SID}$ (E-Q)-ATP	IrtAB$_{\Delta SID}$-ADP
Microscope	FEI Titan Krios			
Magnification	29,000 x	165,000 x		
Voltage (keV)	300			
Electron exposure (e^-/Å^2)	60			
Defocus range (μm)	-1.2 to -1.8			
Pixel size (Å/pixel)	0.82			
Number of movies	1,875	10,551	12,614	2,083
Symmetry imposed	C1			
Final particle images (no.)	143,181	372,559	101,408	44,951
Map resolution (Å)	3.48	2.88	3.12	3.53
FSC threshold	0.143			
Map resolution range (Å)	3.0 – 11.8	2.4 – 9.8	2.7 – 11.8	3.0 – 13.7
**Refinement**				
Initial model used (PDB code)	6TEJ			
Model resolution (Å)	3.2	2.7	2.9	3.2
FSC threshold	0.143			
Model resolution range (Å)	∞ – 3.2	∞ – 2.7	∞ – 2.9	∞ – 3.2
Map sharpening *B* factor (Å^2)	-69.4	-69.4	-77.0	-64.3
Model composition				
Non-hydrogen atoms	8615	8646	8603	8670
Protein residues	1147	1147	1137	1147
Ligands	-	1	5	3
B factors (Å^2)				
Protein	94.43	66.73	52.17	82.03
Ligand	-	79.19	45.11	84.37
R.m.s. deviations				
Bond lengths (Å)	0.004	0.008	0.005	0.004
Bond angles (°)	0.874	0.926	0.849	0.845
Validation				
MolProbity score	2.09	1.99	1.91	2.06
Clash score	9.38	8.27	7.80	9.11
Poor rotamers (%)	0.34	0.23	0.11	0.00
Ramachandran plot				
Favored (%)	88.19	90.20	92.14	89.15
Allowed (%)	11.37	9.28	7.60	10.41
Outliers (%)	0.44	0.52	0.26	0.44
